# Approximate Optimal Deployment of Barrier Coverage on Heterogeneous Bistatic Radar Sensors

**DOI:** 10.3390/s19102403

**Published:** 2019-05-26

**Authors:** Xianghua Xu, Chengwei Zhao, Zongmao Cheng, Tao Gu

**Affiliations:** 1 School of Computer Science and Technology, Hangzhou Dianzi University, Hangzhou 310018, China; zcw@hdu.edu.cn; 2 School of Science, Hangzhou Dianzi University, Hangzhou 310018, China; zmcheng@hdu.edu.cn; 3 Department of Computer Science, RMIT University, Melbourne 3001, Australia; tao.gu@rmit.edu.au

**Keywords:** line barrier coverage, heterogeneous bistatic radar sensor, heterogeneous wireless sensor networks, optimal deployment

## Abstract

Heterogeneous Bistatic Radars (BR) have different sensing ranges and couplings of sensing regions, which provide more flexible coverage for the boundary at complex terrain such as across rivers and valleys. Due to the Cassini oval sensing region of a BR and the coupling of sensing regions among different BRs, the coverage problem of BR sensor networks is very challenging. Existing works in BR barrier coverage focus mainly on homogeneous BR sensor networks. This paper studies the heterogeneous BR placement problem on a line barrier to achieve optimal coverage. 1) We investigate coverage differences of the basic placement sequences of heterogeneous BRs on the line barrier, and prove the optimal basic placement spacing patterns of heterogeneous BRs. 2) We study the coverage coupling effect among adjacent BRs on the line barrier, and determine that different placement sequences of heterogeneous BR transmitters will affect the barrier’s coverage performance and length. The optimal placement sequence of heterogeneous BR barrier cannot be solved through the greedy algorithm. 3) We propose an optimal BRs placement algorithm on a line barrier when the heterogeneous BR transmitters’ placement sequence is predetermined on the barrier, and prove it to be optimal. Through simulation experiments, we determine that the different placement sequences of heterogeneous BR transmitters have little influence on the barrier’s maximum length. Then, we propose an approximate algorithm to optimize the BR placement spacing sequence on the heterogeneous line barrier. 4) As a heterogeneous barrier case study, a minimum cost coverage algorithm of heterogeneous BR barrier is presented. We validate the effectiveness of the proposed algorithms through theory analysis and extensive simulation experiments.

## 1. Introduction

Barrier Coverage is an important sensor placement issue in many industrial, consumer and military applications, such as machine management, health care monitoring, and battlefield surveillance [1]. Recent years have witnessed a trend in which radar sensors have been increasingly deployed to guard militarized zones and monitor warehouse and frontier hazards [2,3,4]. Traditional passive sensors are typically based on a disk sensing model. In contrast, BR sensors use a Cassini oval sensing model [5]. The sensing region of a BR sensor depends on the locations of both BR transmitter and receiver, and is characterized by a Cassini oval. Moreover, since a BR transmitter (or receiver) can potentially form multiple BRs with different BR transmitters (or receivers, respectively), the sensing regions of different BRs are coupled, making the coverage of a BRN great challenge.

Some recent works on BR sensor-based barrier coverage focus mainly on homogeneous BR sensor networks. Gong et al. [6] first studied the optimal placement method for line barrier coverage in BR sensor networks to maximize the worst-case intrusion detectability of the barrier. Wang et al. [7] studied the minimum cost BR sensors deployment problem for long belt barrier. They proposed a line-based equipartition placement strategy to realize long belt barrier coverage with a predefined width and detecting threshold. Yang et al. [8] studied area coverage in BR sensor networks, and proposed an efficient algorithm to solve the Point Coverage Problem which solves the area coverage problem. Chang et al. [9] studied the fault-tolerance deployment problem in BR sensor networks. However, these studies mainly focused on barrier and area coverage in homogeneous BR sensor networks. They assumed that all bistatic radar sensors have the same physical parameters in BR sensor networks, and did not further consider the deployment issue of heterogeneous BR sensors in barrier coverage.

Different from homogeneous BR sensors, heterogeneous BR sensors consist of different types of transmitters with different physical parameters. They transmit different strength radar signal and form coverage areas of different sizes; hence, a BR barrier composed of heterogeneous BR sensors can provide more flexible and practical intrusion protection for boundary at complex terrain, such as across rivers and valleys.

**Figure 1:**
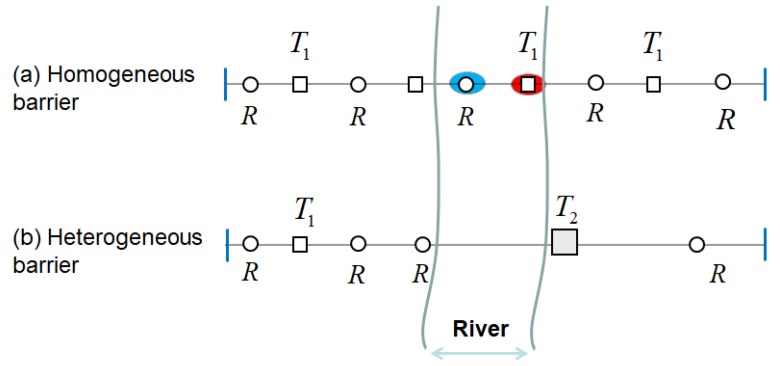
The homogeneous and heterogeneous BR barrier coverage scenario across the same river. (**a**) Homogeneous BR barrier deployment using three *T*_1_ transmitters and five receivers. (**b**) Heterogeneous BR barrier deployment using *T*_1_, *T*_2_ transmitters and four receivers.

For example, Figure 1 illustrates scenarios of deployment homogeneous/heterogeneous BR barrier across the river. We assume that *T*_1_, *T*_2_ are two types of transmitter, and transmitter *T*_2_ sends more strength radar signals than *T*_1_. Therefore, under the same condition of required monitoring threshold, the deployment interval of transmitter *T*_2_ is larger than transmitter *T*_1_. In Figure 1a, in the scenario of homogeneous BR barrier deployment that use three *T*_1_ transmitters and five receivers and can be deployed across rivers, one receiver and one transmitter are place in the river according to *T*_1_ transmitter’s optimal placement interval. Sensors’ deployment in the river will increase additional construction costs of barrier, and placing sensors in rivers are not even allowed in some practical applications. In Figure 1b, we use more powerful BR transmitter *T*_2_ in heterogeneous BR barrier deployment to across the river. Due to the *T*_2_ transmitter has larger deployment interval than *T*_1_, so we can avoid the placement of sensors in the river. Because the unit cost of *T*_2_ transmitter is much more expensive than *T*_1_, so we need to use heterogeneous barrier coverage of *T*_1_ and *T*_2_ to reduce the deployment cost in the practical scenario.

The heterogeneous barrier has more flexible coverage solution adapt to actual deployment practical scenarios than the homogeneous barrier, as demonstrated in Figure 1. However, by applying different types of BR transmitter to construct the heterogeneous BR barrier, different transmitters have different coverage ranges under same sensing signal threshold; hence the basic placement patterns of heterogeneous BR sensors are different from homogeneous ones. Furthermore, due to the fact that a receiver can receive signals from multiple nearby transmitters, there are coverage coupling effects among adjacent different types of BR transmitters and its associated receivers, the combination sequence of different types of BR transmitters on a barrier will affect the barrier coverage range under required sensing signal threshold. It is difficult to find the optimal covering combination of different types of transmitters and receivers on a heterogeneous BR barrier through greedy algorithm to achieve optimal deployment performance. These make the optimal deployment for heterogeneous BR barrier great challenging.

In this paper, we study how to optimize the performance of a heterogeneous BR barrier. For a given numbers of different types of BR transmitters and receivers, we investigate the optimal sensor placement sequences on a line barrier of length *L* to maximize the monitoring performance. 

To the best of our knowledge, this appears the first paper to investigate the optimal sensor deployment problem on heterogeneous BR barrier. The main contributions of our work are summarized as follows.

1. We investigate the coverage differences of the basic placement of different types of BR transmitters and receivers on the line barrier. We find out and prove the optimal basic placement spacing sequences (patterns) of heterogeneous BR sensors on a line. Then, we investigate the coverage coupling effect among adjacent different types of transmitters in the placement sequence on the heterogeneous barrier. Different with homogeneous BR barrier, we discover that placement sequences of different types of BR transmitters on line barrier affect the barrier’s coverage length under same motoring threshold. The optimal placement sequence of heterogeneous BR barrier could not be solved through the greedy algorithm directly.
2. We determine that when the placement orders for heterogeneous transmitters are determined on the barrier, we can construct the optimal BR sensor placement spacing sequence on the line barrier with a predefined monitoring threshold.
3. Through extensive simulation experiments, we determine that the different placement sequences of heterogeneous transmitters on the line barrier have little influence on barrier’s maximum length under a predefined monitoring threshold. We then propose an approximate algorithm to solve the optimal deployment of heterogeneous BR barrier with a very small coverage range of errors and achieve maximum monitoring performance.
4. As a heterogeneous barrier case study, we present a minimum cost coverage algorithm of heterogeneous BR barrier further.
5. Finally, we validate the effectiveness of the proposed algorithms through theoretical analysis and extensive simulation experiments.

The rest of paper is organized as follows: Section 2 reviews the related work. Section 3 introduces the system model and problem description. Section 4 describes the solution of constructing line barrier with approximate optimization monitoring performance. Simulation results are presented in Section 5 and Section 6 concludes the paper.

## 2. Related Works 

Barrier coverage is an important issue in wireless sensor network applications, such as border intrusion detection, environment monitoring and city surveillance [10,11,12,13,14]. In WSN, the sensing model can be classified into passive sensing model [10] and active radar sensing model [6]. Traditional passive sensors are typically based on a disk sensing model. Many researchers have studied barrier coverage problems based on omnidirectional sensing model [1,3,4,10,15,16,17,18,19,20,21,22,23] and directional sector sensing model [2,17,24,25,26]. Different with passive sensors, BR sensors actively transmit radar signals to detect targets. The sensing region of BR sensors depends on the locations of both BR transmitter and receiver, and is characterized by a Cassini oval. Since a BR transmitter (or receiver) can potentially form multiple BRs with different BR transmitters (or receivers, respectively), the sensing regions of different BRs may be coupled, making the coverage of a BR barrier highly non-trivial [6,7]. The barrier coverage problem of bistatic radar sensor network has been studied by some researchers recently [7,8,9,27,28]. Gong et al. [6,27] first studied the optimal placement method for line barrier coverage in BR sensor networks to maximize the worst-case intrusion detectability of the barrier. In [7], the authors studied the minimum cost belt barrier coverage with a predefined width in bistatic radar sensor networks. The authors in [9] studied belt barrier coverage in bistatic radar sensor model. They first proposed a new placement method to reduce the total placement cost and then studied the fault-tolerance deployment issue. The authors in [28] proposed a random Voronoi algorithm to calculate the optimal position of transmitters and receivers so that the maximum distance between all the points of interest in the area to their nearest sensor pair is minimized. In [8], the authors studied area coverage in bistatic radar sensor networks, which is composed of a collection of transmitters and receivers. They proposed an efficient algorithm to solve the Point Coverage Problem, which thus solves the area coverage problem. The existing researches are mainly focus on the barrier and area coverage issue in homogeneous BR sensor networks. They assumed that the bistatic radar sensors have same physical parameters used in the barrier, and did not considered the deployment issue of heterogeneous BR sensors in barrier coverage application.

The heterogeneous sensors have different coverage range and monitoring capabilities, and they are more flexible and practical than homogeneous sensors to meet more applications’ coverage needs. The authors in [18] studied how to form barrier coverage by leveraging multiple types of heterogeneous mobile sensors, and proposed a greedy movement algorithm to efficiently schedule different types of mobile sensors to different gaps while minimizing the total moving cost. In [19], the authors studied the barrier coverage problem in a mobile survivability-heterogeneous wireless sensor network, and proposed a greedy barrier construction algorithm to minimize the barrier’s energy consumption. In [20], the authors realized a heterogeneous barrier-coverage in which guarantees that any intruders are detected by at least one sensor with different sensing capabilities. The authors in [29] characterized the WSN parameters such as node density and sensing range in terms of a desirable detection probability in homogeneous and heterogeneous WSNs, and achieved network connectivity and the corresponding detection probability. In [30], the authors investigated the problem of extending the lifetime of dynamic heterogeneous WSNs with EH sensors to enhancing the total WSN lifetime. The authors in [31] considered the target coverage problem with multiple sensing units in heterogeneous WSNs, and proposed two distributed heuristic schemes to solve the target coverage problem.

The existing research works in BR sensor networks mainly focused on the barrier and area coverage issue in homogeneous BR sensor networks. They assumed that all bistatic radar sensors have the same physical parameters in the BR sensor networks, and did not consider the barrier deployment issue of heterogeneous BR sensors. Different from homogeneous BR sensors, heterogeneous BR sensors can form coverage areas of different sizes, hence the barrier which is composed of heterogeneous BR sensors can provide more flexible and practical intrusion protection for boundary at complex terrain, such as across rivers and valleys.

However, the heterogeneous BR sensors have different coverage ranges and physical coupling effect among BR sensors with different physical sensing parameters, so the placement patterns of heterogeneous BR sensors are different from homogeneous. Furthermore, due to there are coverage coupling effects among adjacent heterogeneous BR sensor pairs, the different marshalling sequence of heterogeneous BR sensors on the barrier will affect the barrier’s coverage performance. These make the optimal construction of heterogeneous BR barrier great challenging. In this paper, we study how to optimize the performance of a heterogeneous BR barrier and minimize its coverage cost. To the best of our knowledge, this is the first paper to study the minimum cost barrier coverage in heterogeneous BR sensor networks.

## 3. Sensor Model and Problem Definition

We define the relevant parameters in Table 1.

### 3.1. BR Sensor Model

In the BR sensor model, the transmitter and sensor can be placed in different positions. Let $T_{i}$ denote transmitter $T_{i}$, $R_{j}$ denote receiver $R_{j}$, $\bar{T_{i}X}$ denote the line segment between transmitter $T_{i}$ and target *X*, and $\left\|T_{i}X\right\|$ denote the Euclidean distance of the line segment $\bar{T_{i}X}$, $\bar{R_{j}X}$ denote the line segment between receiver $R_{j}$ and target *X*, $\left\|R_{j}X\right\|$ denote the Euclidean distance between target *X* and the receiver $R_{j}$.

For a pair of BR sensor $T_{i}-R_{j}$, the signal-to-noise ratio (SNR) of a target *X* can be computed as follows:

(1) $$SNR(X)=\frac{K}{{\left\|T_{i}X\right\|}^{2}\cdot {\left\|R_{j}X\right\|}^{2}}$$

**Figure 2:**
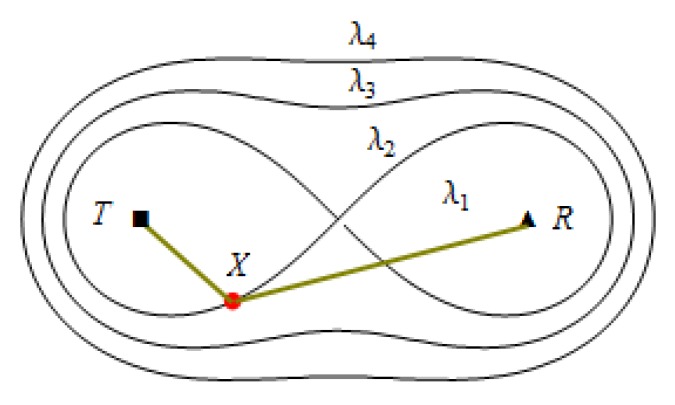
Sensor coverage under different SNR thresholds: $\lambda_{1}<\lambda_{2}<\lambda_{3}<\lambda_{4}$.

Here, K is a constant determined by the physical properties of the BR sensor, $\left\|T_{i}X\right\|$ denotes the Euclidean distance between *T_i_* and *X*, $\left\|R_{j}X\right\|$ denotes the Euclidean distance between $R_{j}$ and *X*. Suppose that λ denotes the SNR threshold of the line barrier, if the target *X* can be detected, then SNR(X)≥λ. When the distance between the sensor changes or the SNR threshold changes, the coverage area will also change, as shown in Figure 2.

We know that a transmitter (receiver) can match multiple receivers (transmitters). In this paper, we consider the placement of line barrier, so for a receiver, we only consider the SNR from the nearest two transmitters [15]. For any target *X* in the barrier area, it can be detected by multiple pairs of BR sensors, and we select the maximum SNR as the SNR of target *X*. At the same time, we introduce the concept of detection values of a target.

**Definition** **1.** *(Detectability): The detectability of a target X, denoted by*I(X)*, is the minimum distance product of X with respect to a BR among all BRs [7]:*

(2) $$I(X)=\underset{\left(T_{i}\in \bar{T},R_{j}\in \bar{R}\right)}{\min}\left\|T_{i}X\right\|\cdot \left\|R_{j}X\right\|$$

*where T_i_ denotes a transmitter, R_j_ denotes a receiver,*$\bar{T}$*denotes the set of transmitters,*$\bar{R}$*denotes the set of receivers,*$\left\|T_{i}X\right\|$*denotes the Euclidean distance between T_i_ and X,*$\left\|R_{j}X\right\|$*denotes the Euclidean distance between R_j_ and X. And*$\bar{T}$*denotes the set of all transmitters,*$\bar{R}$ denotes the set of all receivers.

In combination with SNR threshold λ, we can obtain the detectability threshold of a barrier as follows:

(3) $$I_{\max}=\sqrt{\frac{K}{\lambda }}$$

Thus, if intruder *X* can be monitored when passing through the barrier, then $I(X)\leq I_{\max}$ should be satisfied.

### 3.2. Problem Definition

For a given barrier of length *L* and a numbers of heterogeneous BR transmitters and receivers, we aim to construct a heterogeneous BR barrier to maximize the monitoring performance. We define the problem detailed as Problem 1.

**Problem** **1.** *For q kinds of heterogeneous transmitters T_1_ ~ T_q_, there are the corresponding numbers of heterogeneous transmitters M_1_ ~ M_q_,*$\bar{T}=\left\{\left\{T_{1}^{1},T_{1}^{2},...,T_{1}^{M_{1}}\right\},...,\left\{T_{q}^{1},T_{q}^{2},...,T_{q}^{M_{q}}\right\}\right\}$*, and the numbers of receivers N,*$\bar{R}=\left\{R_{1},R_{2},...,R_{N}\right\}$. *How to construct a heterogeneous BR barrier*P*of length L based on the given set of heterogeneous BR sensors and achieve the optimal detection performance*.

**Figure 3:**
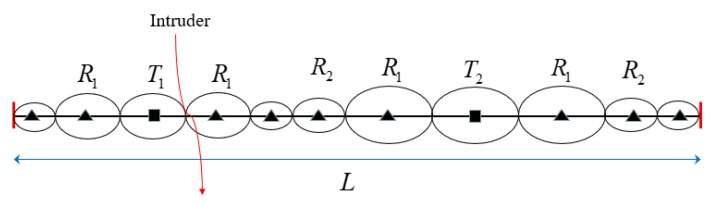
Heterogeneous BR barrier *P* with length *L* to detecting intruders (Squares denote transmitters and triangles denote receivers. *T_1_* and *T_2_* represent two different types of transmitters, and all receivers have the same type.).

As shown in Figure 3, we construct a heterogeneous line barrier *P* with length *L*, in which it consists of two different types of transmitters *T_1_* and *T_2_*, and nine receivers.

For convenience, we define the concept of barrier vulnerability as follows.

**Definition** **2.** *(The vulnerability of barrier): We call the minimum SNR of all points on the barrier the vulnerability of barrier P:*

(4) $$V(P)=\underset{X\in F}{\min}SNR(X)$$

*where X is an arbitrary target point on the line barrier,*F denotes all monitoring locations on the linear barrier P.

Thus, the Problem 1 can be formalized as:

(5) maximize V(P), subject to ‖P‖=L

where ‖P‖ denotes the length of line barrier *P*, *V*(*P*) denotes the vulnerability of the barrier *P*. 

However, we found that the Problem 1 cannot directly obtain its optimal solution. When building a barrier, we need to know the threshold of SNR beforehand, but Problem 1 requires us to get the best threshold of SNR while building a barrier, which is obviously difficult to solve. 

Then, we transform Problem 1 into Problem 2 as follows.

**Problem** **2.** *For q kinds of heterogeneous transmitters T_1_ ~ T_q_, there are the corresponding numbers of heterogeneous transmitters M_1_ ~ M_q,_*$\bar{T}=\left\{\left\{T_{1}^{1},T_{1}^{2},...,T_{1}^{M_{1}}\right\},...,\left\{T_{q}^{1},T_{q}^{2},...,T_{q}^{M_{q}}\right\}\right\}$*, and the numbers of receivers N,*$\bar{R}=\left\{R_{1},R_{2},...,R_{N}\right\}$. *How to build the longest line barrier under a given SNR threshold and a number of heterogeneous sensors*.

The Problem 2 can be formalized as:

(6) $$\begin{array}{l} \mathrm{maximize}\;\left\|P\right\|\\ \mathrm{subject}\;\mathrm{to}\;V(P)\geq \lambda ,\;P\subseteq \left\{\bar{T},\bar{R}\right\} \end{array}$$

where P denotes the barrier which is constructed by set $\left\{\bar{T},\bar{R}\right\}$; this is a sequence of sensors. *V(P)* denotes the vulnerability of the barrier *P*, λ denotes the SNR threshold of line barrier *P* and ‖P‖ denotes the length of line barrier *P*. The optimal barrier’s length of Formula (6) decreases as the SNR threshold increases.

When Problem 2 as Formula (6) was solved, we can design a binary search algorithm to solve Problem 1 as Formula (5), as shown in Algorithm 1. When a set of heterogeneous sensors are given, we first calculate the maximum length of the barrier with the predefine SNR threshold. Then, we can use the binary search Algorithm 1 to approaching the maximum SNR threshold.

The binary search Algorithm 1 to solve Problem 1 is presented as follow:

**Algorithm 1** Compute the optimal deployment for Problem 11. 
$\mathrm{Input}:\bar{T}=\left\{\left\{T_{1}^{1},T_{1}^{2},...,T_{1}^{M_{1}}\right\},...,\left\{T_{q}^{1},T_{q}^{2},...,T_{q}^{M_{q}}\right\}\right\},\bar{R}=\left\{R_{1},R_{2},...,R_{N}\right\},L,\;\omega \left(\mathrm{precision}\;\mathrm{threshold}\right)$
2. 
$\mathrm{Output}:\;\mathrm{the}\;\mathrm{optimal}\;\mathrm{placement}\;\mathrm{order}\;S^{*},\;\mathrm{the}\;\mathrm{optimal}\;\mathrm{value}\;\lambda^{*},l=0,r=INF(a\;\mathrm{larger}\;\mathrm{number})$
3. 
Repeat
4.  $mid\leftarrow \frac{l+r}{2}\;$5.  $\;\mathrm{Call}\;\mathrm{Algorithm}\;2(\;\bar{T},\;\bar{R}\;)\;\mathrm{to}\;\mathrm{get}\;len\;\mathrm{and}\;S\;//\mathrm{order}\;S\;\mathrm{can}\;\mathrm{denote}\;\mathrm{the}\;\mathrm{barrier}\;P$6.   If len>L+ω7.   l←mid8.   Else 9.   r←mid10. 
Until|l−r|≤ω
11. 
$\mathrm{Return}\;S^{*}\leftarrow S,\lambda^{*}\leftarrow mid$


Explanation of Algorithm 1: (1) $\bar{T}$ denotes the set of transmitters needed for the experiment and $\bar{R}$ represents the set of receivers. ω is a precision threshold (a very small number), which is used to deal with the errors in computer representation of floating-point numbers. *L* denotes the length of the barrier required. *r* and *l* denote the upper and lower bounds of the binary search algorithm, where *INF* is a larger number. Sequence *S* represents a sequence of transmitters and receivers, representing a linear barrier *P*. *len* denotes the optimal length of the barrier composed of sequence *S.* (2) Algorithmic idea: It is difficult to directly determine the optimal SNR threshold of the barrier, so we adopt the method of binary search approximation. Firstly, we set the upper and lower bounds *r*, *l* of SNR. Then the expected SNR is set as *mid*, and then the *len* of the linear barrier constructed by the sensor set is calculated when the SNR threshold is *mid*. If *len* is larger than the length *L* of the target barrier, the search is continued in the right half (*mid*, *r*); otherwise, the search is carried out in the left half (*l*, *mid*) until the maximum SNR threshold of the linear barrier constructed by the sensor set is found.

Therefore, how to construct a barrier with approximate optimization monitoring performance is a very critical issue. 

## 4. Constructing Heterogeneous BR Barrier with Approximate Optimal Performance

In this section, we present the solution for constructing heterogeneous barrier with approximate optimal monitoring performance. The brief introduction is as follows.

1. First, we analyze the basic deployment sequences of different types of transmitters and receivers.
  - We divide the barrier as the internal and external edge coverage areas, and discuss the internal area’s placement schema and external edge areas’ placement schema separately;
  - Then, we introduce the basic placement sequence of BR transmitters and receivers, and prove the maximum placement interval on the internal and external coverage areas of heterogeneous transmitters and receivers on the line BR barrier.
2. Based on the above results of basic placement sequence, we investigate Problem 2 which builds the longest line barrier under a given SNR threshold and a numbers of heterogeneous sensors.
  - We consider the effect of combination orders of basic placement sequences of different types of transmitters and receivers to the barrier length. We find that the combination orders of different types of transmitters affect to the barrier length because there have cover coupling effect among different types of adjacent transmitters. It is difficult to find the optimal covering combination of different types of transmitters and receivers on a heterogeneous BR barrier through greedy algorithm to achieve optimal deployment performance.
  - For a predetermined transmitter placement order, we propose an optimal placement algorithm (Algorithm 2) to construct the longest heterogeneous line barrier under a given SNR threshold and a numbers of heterogeneous BR sensors. We prove that Algorithm 2 is optimal placement solution for a predetermined transmitter placement order barrier. But it only partially solves Problem 2.
  - We further investigate the influence of different transmitter placement order on the line barrier coverage length, and validate that the different placement orders of heterogeneous transmitters have a very small coverage range of errors on the barrier length through detailed experiments. Hence, we can use Algorithm 2 to obtain the length of the approximate longest line barrier and the approximate optimal placement scheme of heterogeneous BR sensors given a SNR threshold and a number of heterogeneous sensors. Thus, we solved Problem 2 with an optimal solution under a very small approximation error.
3. Finally, based on Algorithm 2, we can use Algorithm 1 to obtain the approximate optimal monitoring performance of heterogeneous BR barrier through randomly determined placement order of heterogeneous transmitters and binary search.

### 4.1. Maximum Placement Interval Sequence on Homogeneous/Heterogeneous Barrier

#### 4.1.1. Optimal Basic Placement Sequences on Homogeneous Barrier

First, we introduce the relevant content about line barrier. We first treat $H_{l}$ and $H_{r}$ as two virtual nodes and ignore the constant $\left\|H_{l}H_{r}\right\|=L$. Then we place $H_{l}$, $H_{r}$, and all the BR nodes on a horizontal line such that $H_{l}$ and $H_{r}$ are the *leftmost* and *rightmost* nodes, respectively [6].

**Definition** **3.** *(Placement Order and Spacing) [6]: A placement order*S*is an order of all the nodes on the line from left to right:*$S=(H_{l},S_{1},...,S_{J},H_{r})$*, where*J≜M+N*and*$\left(S_{1},...,S_{J}\right)$*is a permutation of the BR nodes such that*$\left\|H_{l}H_{l}\right\|\leq \left\|H_{l}S_{1}\right\|\leq ...\leq \left\|H_{l}S_{J}\right\|\leq \left\|H_{l}H_{r}\right\|$*. The placement spacing*D*of a placement order*S*consists of the distances between neighbor nodes in*S*:*$D≜(\left\|H_{l}S_{1}\right\|,...,\left\|S_{J}H_{r}\right\|)$.

In Definition 3, a series of sensors are placed on a horizontal line to form a line barrier. We can see that the placement sequence can be decomposed into a combination of several basic sequences.

**Definition** **4.** *(The basic placement sequence): In line barrier coverage,*$T-R^{n}\;(T,R_{1},R_{2},...,R_{n})$*is the basic placement sequence (one transmitter and multiple receivers are placed adjacent to each other), and the barrier will be composed of multiple basic placement sequences*.

In Section 3, we have transformed the original problem (Problem 1) into construction of the longest line barrier problem (Problem 2) for a given SNR threshold. 

We introduce the maximum placement spacing between internal coverage area and external coverage area in homogeneous sensor networks. We give the definition of the internal coverage area and external coverage area of line barrier as follows.

**Definition** **5.** *(Internal and External coverage area): In the line barrier coverage, the coverage area between the left-most sensor and the right-most sensor is the internal coverage area, and the rest is the external coverage area*.

**Figure 4:**
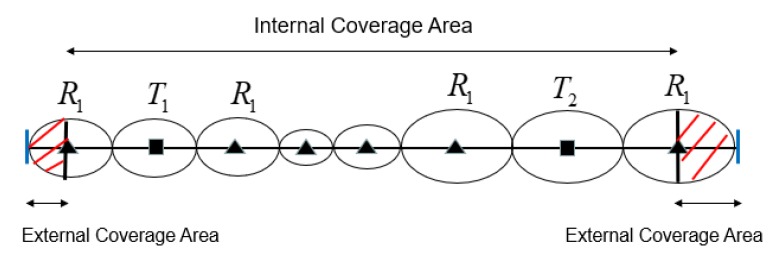
Internal and external coverage area on a BR sensor barrier.

As shown in Figure 4, we can see that the red shaded area is the external coverage area, while the other areas are the internal coverage area. We introduce maximum placement spacing between internal coverage area and external coverage area in the following sections.

##### Optimal Basic Placement Sequences in Internal Coverage Area of Homogeneous Barrier

According to the coverage property of BR sensor model, we can use the basic placement sequence $T-R^{n}$ to deploy a line barrier. In Section 3.1, we get detectability threshold Imax=Kλ. For convenience, Let’s assume $I_{\max}=l\cdot l=l^{2}$. Next, we determine the maximum placement spacing in the internal coverage area of basic placement sequence $T-R^{n}$ and homogeneous placement sequence $T_{1}-R^{n}-T_{1}$ which is the two basic placement sequence that constitutes the BR sensor barrier.

**Theorem** **1.** *For a pair of BR sensors, the maximum detectability is at the midpoint of the segment which connects the transmitter and the receiver [6]*.

**Figure 5:**
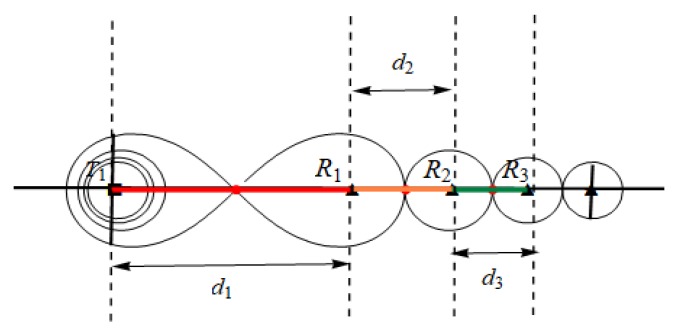
The maximum placement spacing sequence in internal coverage area of sequence $T-R^{n}$. (The length of the red line segment is *d*_1_, the length of the yellow line segment is *d*_2_, and the length of the green line segment is *d*_3_).

As shown in Figure 5, according to Theorem 1, we can get the maximum placement spacing sequence in internal coverage area of sequence $T-R^{n}$ in Theorem 2.

**Theorem** **2.** *For placement sequence*$T-R^{n}$*, the maximum placement spacing sequence in internal coverage area is*$D=\left\{d_{1},d_{2},d_{3},...,d_{n}\right\}$*, where*$d_{i}=2(\sqrt{i}-\sqrt{i-1})l,\;i=1,2,...,n$*,*n is the number of receivers.

Proof: The proof of Theorem 2 is in the appendix. 

**Figure 6:**
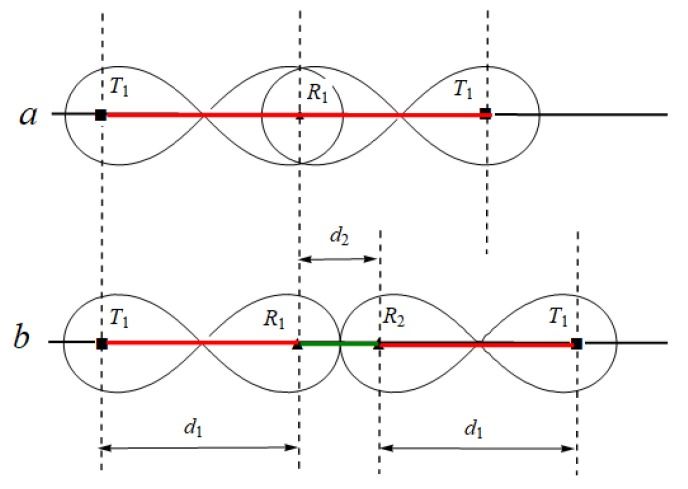
The maximum placement spacing sequence in internal coverage area of sequence $T_{1}-R^{n}-T_{1}$. (**a**) when *n* is odd; (**b**) when *n* is even.

Next, we discuss the maximum placement spacing sequence in internal coverage area of homogeneous placement sequence $T_{1}-R^{n}-T_{1}$. We know that homogeneous sequence $T_{1}-R^{n}-T_{1}$ uses the same type of transmitter, so coverage sequence has symmetry. We have obtained the maximum placement spacing sequence in internal coverage area of sequence $T-R^{n}$, so we can obtain the maximum placement spacing sequence in internal coverage area of sequence $T_{1}-R^{n}-T_{1}$ by this property. As shown in Figure 6, we can get the maximum placement spacing sequence in internal coverage area of $T_{1}-R^{n}-T_{1}$ in Theorem 3.

**Theorem** **3.** *For placement sequence*$T_{1}-R^{n}-T_{1}$, *the maximum placement spacing sequence in internal coverage area is*

1. *When*n*isodd,*$D=\left\{d_{1},d_{2},...,d_{(n+1)/2},d_{(n+1)/2},...,d_{2},d_{1}\right\}$.
2. *When*n*is even,*$D=\left\{d_{1},d_{2},...,d_{n/2+1},...,d_{2},d_{1}\right\}$.
  *Where*$d_{i}=2(\sqrt{i}-\sqrt{i-1})l,\;i=1,2,...,n$*,*n is the number of receivers.

Proof: The proof of Theorem 3 is in the appendix. 

Optimal Basic Placement Sequences in External Coverage Area of Homogeneous Barrier

Next, we determine the maximum placement spacing sequence on the external coverage area of basic placement sequence $T-R^{n}$ and homogeneous placement sequence $T_{1}-R^{n}-T_{1}$.

**Figure 7:**
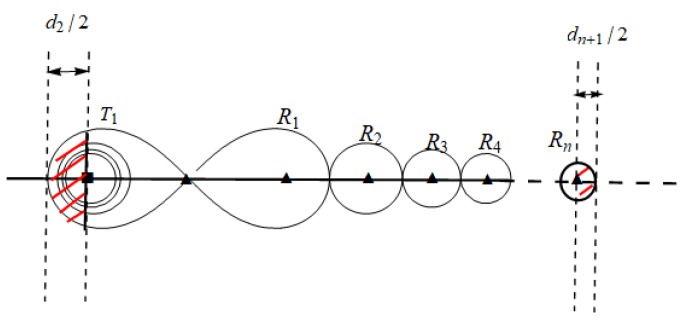
The maximum placement spacing sequence in external coverage area of sequence $T-R^{n}$.

According to symmetry and the proof of Theorem 1, we can easily get the maximum placement spacing sequence in the external coverage area of sequence $T-R^{n}$ in Theorem 4, as shown in Figure 7.

**Theorem** **4.** *For homogeneous BR placement sequence*$T-R^{n}$*, the maximum placement spacing sequence in external coverage area is*$E=(EL,ER)=(\frac{d_{2}}{2},\frac{d_{n+1}}{2})$*, where*$d_{i}=2(\sqrt{i}-\sqrt{i-1})l,\;i=1,2,...,n$*,*n is the number of receivers.

Proof: The proof of Theorem 4 is in the appendix.

**Figure 8:**
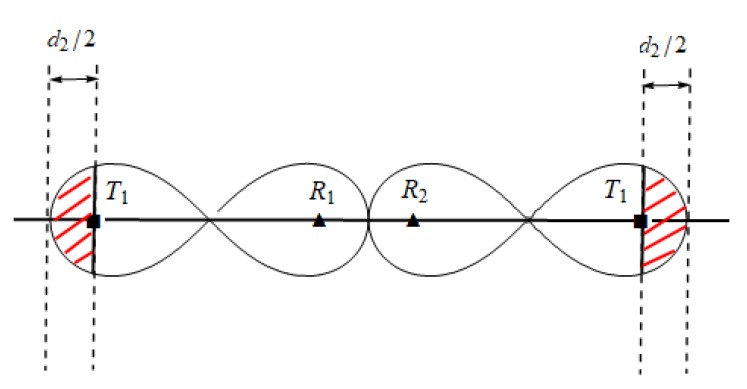
The maximum placement spacing sequence in external coverage area of sequence $T_{1}-R^{n}-T_{1}$.

Then, as show in Figure 8, we can get the maximum placement spacing sequence in external coverage area of sequence $T_{1}-R^{n}-T_{1}$ in Theorem 5 through similar thinking and calculation.

**Theorem** **5.** *For homogeneous BR placement sequence*$T_{1}-R^{n}-T_{1}$*, the maximum placement spacing sequence in external coverage area is*$E=(EL,ER)=(\frac{d_{2}}{2},\frac{d_{2}}{2})$*, where*$d_{2}=2(\sqrt{2}-1)l$.

Proof: The proof of Theorem 5 is in the appendix.

#### 4.1.2. Optimal Basic Placement Sequences on Heterogeneous Barrier

Heterogeneous BR sensor placement sequences are mainly composed of several different basic BR sequences, such as basic sequences $T_{1}-R^{n}$ and $T_{2}-R^{n}$, which can be combined into heterogeneous sequences $T_{1}-R^{n}-T_{2}$. In homogeneous sequence $T_{1}-R^{n}-T_{1}$, the sensor coverage area is symmetrical, which makes the placement of transmitter very convenient. But in heterogeneous sequence $T_{1}-R^{n}-T_{2}$, because of the different physical parameter of the two transmitters, the SNR of radar transmitter’s signal intensity is different. For each receiver, we need to consider the combination relationship between the two transmitters and get the optimal placement position. In this section, we will discuss the maximum placement spacing of heterogeneous sequence $T_{1}-R^{n}-T_{2}$ in details.

##### Optimal Basic Placement Sequences in Internal Coverage Area of Heterogeneous Barrier

In this section, we determine the maximum placement spacing interval in internal coverage area of heterogeneous placement sequence $T_{1}-R^{n}-T_{2}$. $T_{1}$, $T_{2}$ are two heterogeneous transmitters, in Section 4.1.1, we determined that the maximum placement spacing sequence of basic sequence $T-R^{n}$ is $D=\left\{d_{1},d_{2},d_{3},...,d_{n}\right\}$. However, we notice that if the sensor’s physical properties change, the maximum placement spacing will change as well. As a result, the maximum placement spacing interval of sequence $T_{1}-R^{n}$ is different from sequence $T_{2}-R^{n}$. Here we set the maximum placement spacing interval of sequence $T_{1}-R^{n}$ is $D^{1}=\left\{d_{1}^{1},d_{2}^{1},d_{3}^{1},...,d_{n}^{1}\right\}$, while the maximum placement spacing interval of sequence $T_{2}-R^{n}$ is $D^{2}=\left\{d_{1}^{2},d_{2}^{2},d_{3}^{2},...,d_{n}^{2}\right\}$, and the threshold of detectability determined by transmitter $T_{1}$ is larger than which is determined by transmitter $T_{2}$. 

We can get the maximum placement spacing sequence in internal coverage area of sequence $T_{1}-R_{n}-T_{2}$ in Theorem 6. 

Considering the importance of heterogeneous placement sequence $T_{1}-R^{n}-T_{2}$, we analyze and prove it directly as follow.

**Theorem** **6.** *For heterogeneous BR placement sequence* $T_{1}-R^{n}-T_{2}$ *, the maximum placement spacing sequence in internal coverage area is*

1. *When* $d_{n+1}^{1}\leq d_{1}^{2}$ *,* $D=\left\{\max(d_{1}^{1},d_{n+1}^{2}),\max(d_{2}^{1},d_{n}^{2}),...,\max(d_{n}^{1},d_{2}^{2}),\max(d_{n+1}^{1},d_{1}^{2})\right\}$
2. *When*$d_{n+1}^{1}>d_{1}^{2}$*,*$D=\left\{d_{1}^{1},d_{2}^{1},...,d_{n}^{1},d_{n+1}^{1}/2+\frac{l_{2}^{2}}{d_{n+1}^{1}/2}\right\}$.
  *Where*$d_{i}^{1}=2(\sqrt{i}-\sqrt{i-1})l_{1},\;d_{i}^{2}=2(\sqrt{i}-\sqrt{i-1})l_{2},\;i=1,2,...,n$*,*n*is the number of receivers*.

**Proof.**  For heterogeneous BR placement sequence $T_{1}-R^{n}-T_{2}$, we also discuss the different cases in which the number of receivers ranges from 1 to n.

**Figure 9:**
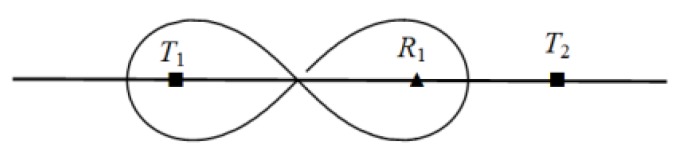
Maximum placement spacing of heterogeneous BR sequence $T_{1}-R^{n}-T_{2}$ when n=1.

When n=1, as shown in Figure 9.

**Figure 10:**
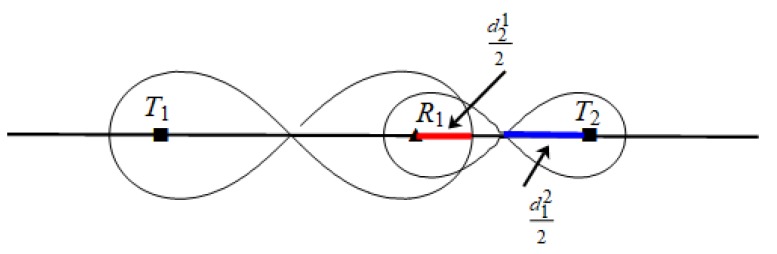
Maximum placement spacing of heterogeneous BR sequence $T_{1}-R^{n}-T_{2}$ when $d_{2}^{1}\leq d_{1}^{2}$. ($d_{2}^{1}$ denotes the maximum deployment interval between *R*_1_ and *R*_2_ in the *T*_1_–*R*_1_–*R*_2_ sequence, and $d_{1}^{2}$ represents the maximum deployment interval between *T*_2_ and *R*_1_ in the *T*_2_–*R*_1_ sequence).

At this point, $\left\|T_{1}R_{1}\right\|=d_{1}^{1}$. However, because the physical properties of transmitter $T_{1}$ and transmitter $T_{2}$ are different, the size of these two coverage areas is different as well and needs to be analyzed in the following two cases:

*1.* When $\frac{d_{2}^{1}}{2}\;\leq \;\frac{d_{1}^{2}}{2}$, as shown in Figure 10.

At this point, $\left\|T_{1}R_{1}\right\|=d_{1}^{1}$*,* $\left\|T_{2}R_{1}\right\|=d_{1}^{2}$ we can find $d_{1}^{2}$ is the maximum distance for line segment $\bar{T_{2}R_{1}}$.

**Figure 11:**
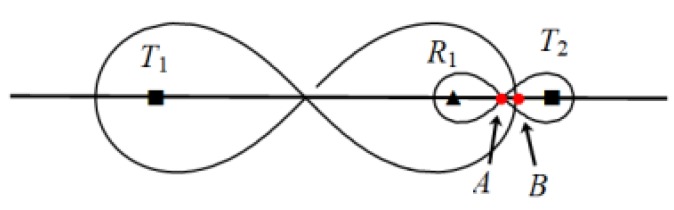
The placement spacing of heterogeneous BR sequence $T_{1}-R^{n}-T_{2}$ when $d_{2}^{1}>d_{1}^{2}$.

*2.* When $\frac{d_{2}^{1}}{2}\;>\;\frac{d_{1}^{2}}{2}$*,* as shown in Figure 11.

**Figure 12:**
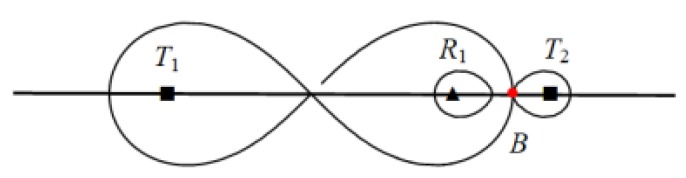
Maximum placement spacing of heterogeneous sequence $T_{1}-R^{n}-T_{2}$ when $d_{2}^{1}>d_{1}^{2}$.

At this point, $\left\|R_{1}B\right\|=\frac{d_{2}^{1}}{2}$, and $\left\|R_{1}B\right\|>\left\|R_{1}A\right\|$, but we can see that the line coverage between transmitter $T_{2}$ and receiver $R_{1}$ can continue to increase, as shown in Figure 12:

Under the given SNR threshold, the threshold of detectability of transmitter $T_{1}$ is $I_{\max}^{1}=l_{1}\cdot l_{1}=l_{1}^{2}$, and the threshold of detectability of transmitter $T_{2}$ *is* $I_{\max}^{2}=l_{2}\cdot l_{2}=l_{2}^{2}$. At this point, the detectability of point B *is* $I_{\max}^{2}$*,* so there has $\left\|T_{2}B\right\|\cdot \left\|R_{1}B\right\|=l_{2}^{2}$*, then* $\left\|T_{2}B\right\|=\frac{l_{2}^{2}}{d_{2}^{1}/2}$. As a result, $\left\|T_{1}R_{1}\right\|=d_{1}^{1}$*,* $\left\|T_{2}R_{1}\right\|=d_{2}^{1}/2+\frac{l_{2}^{2}}{d_{2}^{1}/2}$, simple proof can be used to determine the current placement as the best placement.

When n≥2*,* we could placement sensors in the same way. □

##### Optimal Basic Placement Sequences in External Coverage Area of Heterogeneous Barrier

The heterogeneous placement sequence $T_{1}-R^{n}-T_{2}$ has two different types of transmitter, because of the different physical properties of transmitter, the coverage areas which is composed of BR sensors is different. Hence in this case, we cannot simply adopt the method of Section 4.1.1 to get the maximum placement spacing interval in external coverage area of heterogeneous placement sequence $T_{1}-R^{n}-T_{2}$. In Section 4.1.2, we analyzed the maximum placement spacing interval in internal coverage area of placement sequence $T_{1}-R^{n}-T_{2}$, and we now analyze its maximum placement spacing interval in external coverage area based on this. Similarly, we will consider it in the following two cases, $d_{2}^{1}\leq d_{1}^{2}$ and $d_{2}^{1}>d_{1}^{2}$.

We can get the maximum placement spacing sequence in external coverage area of sequence $T_{1}-R_{n}-T_{2}$ in Theorem 7. The proof of this part is similar to Theorem 6. We also give the proof directly as follow.

**Theorem** **7.** *For heterogeneous BR placement sequence*$T_{1}-R^{n}-T_{2}$, *the maximum placement spacing sequence in external coverage area is*

$$\{\begin{array}{ll} E=(\frac{d_{2}^{1}}{2},\frac{d_{2}^{2}}{2}), & \mathrm{when}\;d_{n+1}^{1}\leq d_{1}^{2},\\ E=\left(\frac{d_{2}^{1}}{2},\sqrt{l_{2}^{2}+{\left(\frac{d_{n+1}^{1}}{4}+\frac{l_{2}^{2}}{d_{n+1}^{1}}\right)}^{2}}-\left(\frac{d_{n+1}^{1}}{4}+\frac{l_{2}^{2}}{d_{n+1}^{1}}\right)\right), & \mathrm{when}\;d_{n+1}^{1}>d_{1}^{2}, \end{array}$$

*where*$d_{i}^{1}=2(\sqrt{i}-\sqrt{i-1})l_{1},\;d_{i}^{2}=2(\sqrt{i}-\sqrt{i-1})l_{2},\;i=1,2,...,n$*,*n is the number of receivers.

**Proof.**  For heterogeneous BR placement sequence $T_{1}-R^{n}-T_{2}$*,* we also discuss the different cases in which the number of receivers ranges from 1 to *n*.

**Figure 13:**
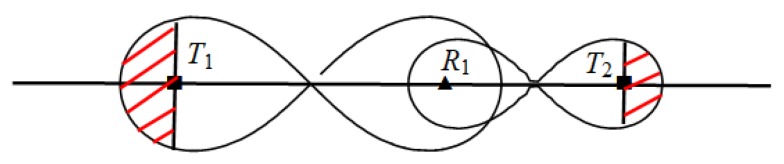
External coverage area of heterogeneous BR sequence $\left(T_{1},T_{2}\right)$ when $d_{2}^{1}\leq d_{1}^{2}$.

1. When $d_{2}^{1}\leq d_{1}^{2}$*,* as shown in Figure 13:

We can obtain the maximum placement spacing interval in the external coverage area, that is $E=(\frac{d_{2}^{1}}{2},\frac{d_{2}^{2}}{2})$. At this time, when the number of receivers between $T_{1}$ and $T_{2}$ increases to n*,* the maximum placement spacing interval in external coverage area is $E=(\frac{d_{2}^{1}}{2},\frac{d_{2}^{2}}{2})$.

**Figure 14:**
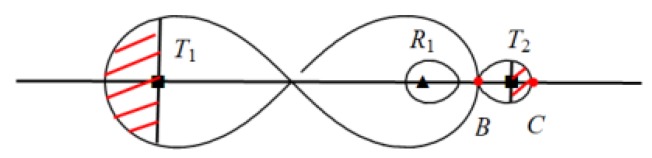
External coverage area of heterogeneous BR sequence $\left(T_{1},T_{2}\right)$ when $d_{2}^{1}>d_{1}^{2}$.

2. When $d_{2}^{1}>d_{1}^{2}$, as shown in Figure 14:

We have $\left\|R_{1}B\right\|=\frac{d_{2}^{1}}{2}$ and $\left\|T_{2}B\right\|=\frac{l_{2}^{2}}{d_{2}^{1}/2}$, because the detectability of point C is $I_{\max}^{2}$, so $\left\|T_{2}C\right\|\cdot \left\|R_{1}C\right\|=l_{2}^{2}$*,* substituting the known value into the above formula can obtain $\left\|T_{2}C\right\|=\sqrt{l_{2}^{2}+{\left(\frac{d_{2}^{1}}{4}+\frac{l_{2}^{2}}{d_{2}^{1}}\right)}^{2}}-\left(\frac{d_{2}^{1}}{4}+\frac{l_{2}^{2}}{d_{2}^{1}}\right)$. When the number of receivers is n*,* and $d_{n+1}^{1}>d_{1}^{2}$*,* the sensor coverage is similar to Figure 14, that is $\sqrt{l_{2}^{2}+{\left(\frac{d_{n+1}^{1}}{4}+\frac{l_{2}^{2}}{d_{n+1}^{1}}\right)}^{2}}-\left(\frac{d_{n+1}^{1}}{4}+\frac{l_{2}^{2}}{d_{n+1}^{1}}\right)$. Therefore, when $d_{n+1}^{1}>d_{1}^{2}$*,* the length of the external coverage area of placement sequence $T_{1}-R^{n}-T_{2}$ is *:* $E=\left(\frac{d_{2}^{1}}{2},\sqrt{l_{2}^{2}+{\left(\frac{d_{n+1}^{1}}{4}+\frac{l_{2}^{2}}{d_{n+1}^{1}}\right)}^{2}}-\left(\frac{d_{n+1}^{1}}{4}+\frac{l_{2}^{2}}{d_{n+1}^{1}}\right)\right)$. □

So, we get Theorem 7.

According to Theorem 7, we determine that compared with the external coverage area of homogeneous BR placement sequence, the length of the external coverage area of heterogeneous BR placement sequence varies with the physical parameters of adjacent transmitters. 

Therefore, for the external coverage area, we need to consider separately under different circumstances. 

### 4.2. Effects of Different Combination Orders of Basic Placement Sequences on Barrier Length

In previous Section 4.1.1 and Section 4.1.2, we have solved the maximum placement spacing interval of heterogeneous BR basic placement sequence. Based on the above basic placement sequence, we further investigate Problem *2* as described in Formula (6), how to construct the longest line barrier with the predefined SNR threshold when a set of heterogeneous BR sensors are given. 

Firstly, we analyze the influence of different combinations order of different types of transmitters on the barrier to its covered length. 

Then, we determine that under the given placement order of heterogeneous transmitters, we can construct an optimal solution for Problem 2 and achieve the longest barrier deployment. 

Finally, we investigate the influence of different transmitter placement order to the barrier length. And we determine that the heterogeneous transmitters’ different placement order will affects the barrier length, but the variation of barrier length caused by different combinations of transmitters place order on the barrier is very small relative to the barrier’s length. 

Different with the construction of homogeneous line barrier: in the construction of heterogeneous barrier, there are much more possible combinations of transmitter-receiver-transmitter pairs as basic sensor placement patterns, as $T_{i}-R^{n}-T_{j}$, where i, j={1,...,M}. Furthermore, the different combination orders of these basic placement sequences on a barrier have the adjacent coupling effect on the coverage ranges, the optimal BR placement sequence of heterogeneous barrier cannot be solved by greedy algorithm directly.

#### 4.2.1. Differences of Basic Placement Sequence of Heterogeneous Transmitters $T_{i}-R^{n}-T_{j}$

**Figure 15:**
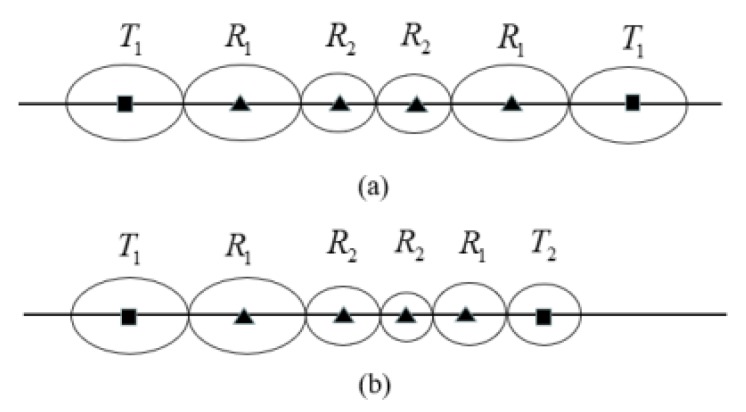
Differences of basic placement sequence homogeneous/heterogeneous. (**a**) Homogeneous basic placement sequences; (**b**) Heterogeneous basic placement sequences.

In homogeneous barrier, basic placement sequence $T_{1}-R^{n}-T_{1}$ is mainly used, as shown in Figure 15a. We can see that sequence $T_{1}-R^{n}-T_{1}$ is symmetrical, which means that we only need to determine the location of half of the receivers to determine the placement of all sensors in the whole sequence. The symmetry property can easily determine the coverage length of sequence $T_{1}-R^{n}-T_{1}$.

In heterogeneous barriers, there are many possible combinations of heterogeneous transmitter-receiver-transmitter pairs as basic BR placement patterns $T_{i}-R^{n}-T_{j}$, where i, j={1,...,M}. Thus, determining the optimal placement spacing of receivers in the basic sensor placement patterns on a heterogeneous BR barrier is more complicated than with a homogeneous BR barrier.

For example, for three types of transmitters: $\left\{T_{1},T_{2},T_{3}\right\}$, there are six type of basic BR placement sequence $\left\{T_{1},R^{n},T_{2}\},\{T_{2},R^{n},T_{1}\},...,\{T_{2},R^{n},T_{3}\}\{T_{3},R^{n},T_{2}\right\}$. As shown in Figure 15b, because the physical properties of transmitters $T_{1}$ and $T_{2}$ are different, the coverage range of $T_{1}-R^{4}-T_{2}$ is asymmetric. For each receiver in sequence $T_{1}-R^{n}-T_{2}$, we need to determine the coverage of BR sensor pairs formed with $T_{1}$ and $T_{2}$ separately. 

##### Different Combination Orders of Basic Placement Sequence $T_{i}-R^{n}-T_{j}$

**Figure 16:**
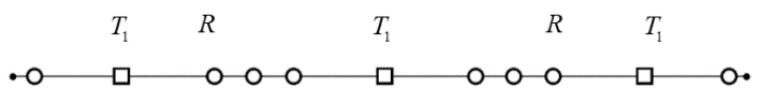
Transmitter and receiver placement sequence in homogeneous line BR barrier (Squares denote transmitters and circles denote receivers).

In homogeneous line barrier coverage, we can construct the optimal line barrier by using multiple identical basic placement sequences $T_{1}-R^{n}-T_{1}$. For example, in [6,7], the authors use similar two basic placement sequences: $\text{\{}T_{1}-R^{3}-T_{1}\text{\}},\text{\{}T_{1}-R^{3}-T_{1}\}$, as shown in Figure 16 (Gong [6] proposed the optimal basic placement in homogeneous BR). In homogeneous networks, transmitters have the same physical attributes and coverage ranges. The coverage of basic placement sequences $T_{1}-R^{3}-T_{1}$ is symmetrical. This makes the construction of placement sequences in homogeneous BR barrier relatively easy.

However, for *q* types of transmitters, there are many different combinations of basic placement sequences of $T_{i}-R^{n}-T_{j}$.On the heterogeneous line barrier coverage, the different combinations order of different basic placement sequences has coupling effect on the barrier length. 

**Figure 17:**
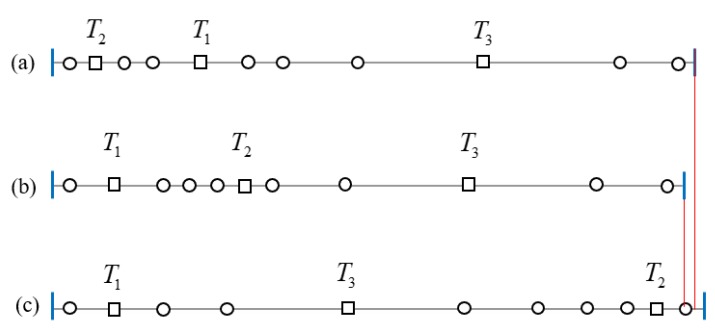
The barrier length’s variation of three optimal BR sensor placement sequences on the heterogeneous barrier (Squares denote transmitters and circulars denote receivers): (**a**) optimal placement sequence: $\left(RT_{2}RRT_{1}RRRT_{3}RR\right)$; (**b**) optimal placement sequence: $\left(RT_{1}RRRT_{2}RRT_{3}RR\right)$; (**c**) optimal placement sequence: $\left(RT_{1}RRT_{3}RRRRT_{2}R\right)$.

As shown in Figure 17, we use the same set of sensors to construct three barriers, and observe the difference of the length of the linear barrier by comparing different combinations of sensors.

There are three heterogeneous transmitters and eight receivers. For the three combination orders of transmitters: $\left(T_{2},T_{1},T_{3}\right)$, $\left(T_{1},T_{2},T_{3}\right)$ and $\left(T_{1},T_{3},T_{2}\right)$, we construct the corresponding heterogeneous barrier placement sequences by greedy search: $\left(RT_{2}RRT_{1}RRRT_{3}RR\right)$,$\left(RT_{1}RRRT_{2}RRT_{3}RR\right)$ and $\left(RT_{1}RRT_{3}RRRRT_{2}R\right)$, as shown in Figure 17a–c. Figure 17 reveals that the different combination orders of heterogeneous transmitters affect the coverage length on the heterogeneous BR barrier. 

Therefore, when constructing heterogeneous BR barrier, the combination of different basic placement sequences will become much more complex. If we adopt an exhaustive method, we can consider all the situations, the algorithm’s time complexity is O(m!).

Therefore, we need to further investigate the approximate algorithm of optimal placement sequence on heterogeneous BR barrier. We discuss the problem in the following two steps:

(1) Firstly, for simplification, we consider how to construct the longest barrier when the all transmitters placement order is determined.
(2) Then we investigate the influence of different placement orders of transmitters on the barrier’s coverage length and design an approximate algorithm of optimal placement sequence on heterogeneous line BR barrier with very small approximate accuracy error.

#### 4.2.2. Construction of Longest Barrier When Heterogeneous Transmitters Placement Order Is Determined

**Figure 18:**
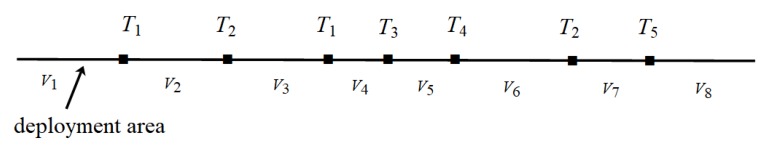
The transmitter placement order and their receivers’ placement areas.

In this section, we will consider how to construct the longest line barrier when the placement order of all transmitters is determined. We have five different transmitter types: $T_{1}$~ $T_{5}$. As shown in Figure 18, $\left\{T_{1},T_{2},T_{1},T_{3},T_{4},T_{2},T_{5}\right\}$, the placement order of the heterogeneous transmitters is given. On this basis, in order to build the longest barrier, we only need to determine the location of the receivers. In Figure 18, at this point it is determined how to deploy the receivers in the eight placement areas ($V_{1}$~$V_{8}$) reasonably so that the length of the barrier achieves the maximum. In order to meet the actual situation, we first placed a receiver in all the eight areas ($V_{1}$~$V_{8}$) to construct an available initial barrier.

Next, we consider the placement of rest receivers on the barrier. Our goal is to realize the longest barrier length as long as possible. For each receiver, it should be placed at the position where the length of the barrier can be increased as long as possible. In Section 4.1, we define the internal coverage area and the external coverage area, so we consider inserting the receiver into the internal coverage area ($V_{2}∼V_{7}$) and the external coverage area ($V_{1}$,$V_{8}$), respectively.

**Theorem** **8.** *In the internal coverage area, if a receiver is inserted into any sequence*$T_{i}-R^{n}-T_{j}$, *the coverage length of other sequences in the internal coverage area will not be affected*.

**Figure 19:**
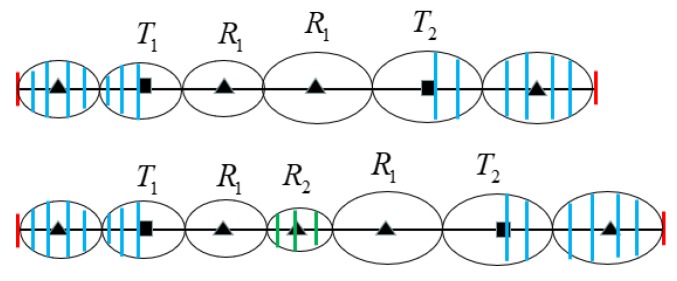
When a receiver is inserted into sequence $T_{1}-R^{n}-T_{2}$.

**Proof of Theorem** **8.** In Section 4.1, we prove and give the maximum placement spacing interval of the sensors, so in the optimal placement strategy, we will use this maximum placement spacing sequence to place the sensor. When we insert a receiver into sequence $T_{i}-R^{n}-T_{j}$*,* we will select the appropriate placement spacing interval in sequence D through specific calculation. This only increases the coverage length of current sequence $T_{i}-R^{n}-T_{j}$*,* and does not affect the coverage length between adjacent sequences in internal coverage area. As shown in Figure 19, when a receiver is inserted into Sequence $T_{1}-R^{2}-T_{2}$*,* only the coverage length of Sequence $T_{1}-R^{2}-T_{2}$ is increased (the coverage length of $R_{2}$ shadow part is increased), and the coverage area outside Sequence $T_{1}-R^{2}-T_{2}$ (blue shadow area) is not affected. □

(1) For the internal coverage area, in Theorem 8, we know that if a receiver is inserted into any sequence $T_{i}-R^{n}-T_{i+1}$, the coverage length of other sequences in the internal coverage area will not be affected. So, for a receiver R, we choose to place it into the placement area ($V_{2}∼V_{7}$) where the length of the barrier increases as long as possible. The increase of the barrier’s length when receiver R is inserted into a placement area can be figured out by the maximum placement spacing interval methods in internal coverage area which are given in Section 4.1.1 and Section 4.1.2.
(2) For the external coverage area, in Figure 17, we need to place a receiver in placement areas $V_{1}$ and $V_{8}$. This situation is relatively simple. We only need to calculate the increased barrier length according to the maximum placement spacing interval method given in Section 4.1.1 and Section 4.1.2.

To sum up, we find that this problem can be solved by a greedy algorithm. The following is a greedy algorithm for constructing the longest line barrier when the transmitters are placed in order.

**Algorithm 2** Build the longest barrier when heterogeneous transmitters’ order is given.12.
$\mathrm{Input}:\;\bar{T}=\left\{\left\{T_{1}^{1},T_{1}^{2},...,T_{1}^{M_{1}}\right\},...,\left\{T_{q}^{1},T_{q}^{2},...,T_{q}^{M_{q}}\right\}\right\},\;\bar{R}=\left\{R_{1},R_{2},...,R_{N}\right\}$
13. 
Output: the optimal length of barrier len, the optimal BR placement order υ
14. 
$\sigma =\phi ,\tau =\phi ,\upsilon =\phi ,len=0,\;m=M_{1}+...+M_{q}$
15. 
$//\;\mathrm{Random}\;\mathrm{generation}\;\mathrm{of}\;\mathrm{transmitter}\;\mathrm{sequence}\;S_{T}\mathrm{with}\;m\;\mathrm{transmitters}\prime \;\mathrm{set}\;\bar{T}$
16. 
$S_{T}\leftarrow \;\mathrm{Random}(\;\bar{T})\;$
17. 
// Place m + 1 receivers into m + 1 segment areas (place one receiver per sengment)
18. 
$//\;\mathrm{to}\;\mathrm{construct}\;\mathrm{an}\;\mathrm{initial}\;\mathrm{barrier}\;\tau \;\mathrm{for}\;S_{T}\;$
19. 
$\tau \leftarrow \mathrm{InitBarrier}(S_{T})\;$
20. 
len=Length(τ) // Initial length len of the initial barrier
21. 
For i←1:N−m−1
22.   For j←1:m+123.   $\;\mathrm{Compute}\;\mathrm{the}\;\mathrm{location}\;\mathrm{of}\;\mathrm{receiver}\;p_{ij}\mathrm{when}\;\mathrm{placing}\;\mathrm{one}\;\mathrm{receiver}\;\mathrm{in}\;\mathrm{sequence}\;S_{T}$24.   $\;\mathrm{Compute}\;\mathrm{the}\;\mathrm{increment}\;\mathrm{of}\;\mathrm{the}\;\mathrm{barrier}\text{'}s\;\mathrm{length}\;L_{ij}\;$25.   $\sigma \leftarrow \sigma \cup \left\{L_{ij}\right\}$26.   $\tau \leftarrow \tau \cup \left\{p_{ij}\right\}$27.   End For28.  $L^{*}\leftarrow \max(\sigma ),\;p^{*}\leftarrow \tau .\mathrm{index}(L^{*})$29.  $len\leftarrow len+L^{*},\;\upsilon \leftarrow \upsilon \cup \left\{\;\tau \left(p^{*}\right)\;\right\}$30.  σ.clear(), τ.clear()31. 
End For
32. 
Return (len, υ)


Explanation of Algorithm 2: 

(1) σ denotes the set of increments in barrier length when the same transmitter is placed in different locations and τ denotes the set of locations. $L^{*}$ denotes the maximum increase in the length of the linear barrier by placing a receiver at a time and $p^{*}$ denotes the best location at this time. The set of transmitters is $\bar{T}=\left\{\left\{T_{1}^{1},T_{1}^{2},...,T_{1}^{M_{1}}\right\},...,\left\{T_{q}^{1},T_{q}^{2},...,T_{q}^{M_{q}}\right\}\right\}$, the set of receivers is $\bar{R}=\left\{R_{1},R_{2},...,R_{N}\right\}$. *S_T_* represents a sequence of transmitters randomly generated from set $\bar{T}$.

(2) Algorithmic idea: First, we randomly determine the placement sequence $S_{T}$ of the *m* ($m=M_{1}+...+M_{q}$) transmitters: $S_{T}\leftarrow \;\mathrm{Random}(\;\bar{T})\;$, and then it forms m+1 placing segment areas as $V_{1}$~$V_{m+1}$. In order to meet the actual situation, we first place m+1 receivers into segment areas to construct an initial barrier τ for $S_{T}$ (one receiver per segment area): $\tau \leftarrow \mathrm{InitBarrier}(S_{T})$, which the initial barrier’s length is len. For the remaining N−m−1 receivers, we place each receiver in different areas in turn, and calculate the increment of the barrier length at each time. We choose the position where the maximum increment is placed for the receiver, so as to get the final linear barrier.

#### 4.2.3. Effect of Different Placement Order of Heterogeneous Transmitters on BR Barrier Length

In Section 4.2.2, we propose the construction algorithm (Algorithm 2) of the longest line barrier when the transmitter placement order is determined. In addition, we also need to consider the effect of different transmitter placement order on the line barrier length.

It is difficult to quantitatively describe the effect of different transmitter placement orders on the length of line barrier in theory, so the experimental method is used to observe the relationship between them. We take the transmitter into six different types, one for each type. In the case of given the SNR threshold, the detectability thresholds of the pairs of sensors which are formed by the 6 types of transmitters and receivers are {4, 36, 81, 169, 324, 625}, respectively, while the number of receivers is three hundred.

**Figure 20:**
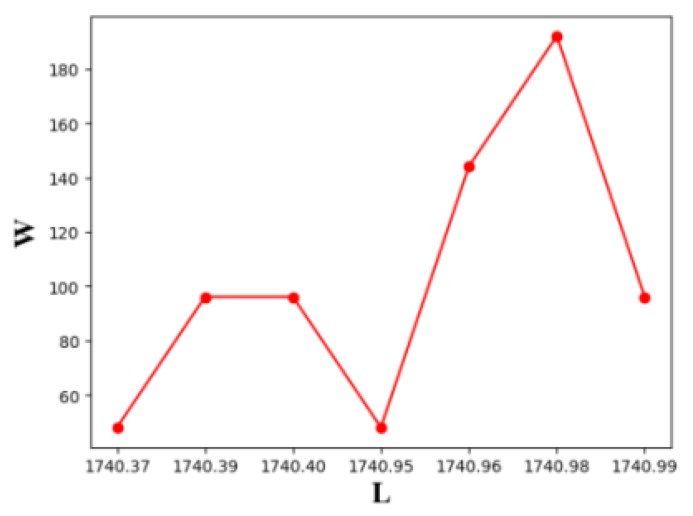
The influence of different placement sequences of heterogeneous transmitter on the length of BR barrier (We use 6 types of transmitters, one for each type, and test on 720 heterogeneous BR placement sequences).

For the given set of sensors, we enumerate all the transmitter placement orders $S(S_{1}∼S_{720})$, a total number of 720, and use Algorithm 2 to calculate the optimal barrier length under different transmitter placement sequences, as shown in Figure 20.

L denotes the length of the barrier and W is the number of line barriers of a certain length (the number of transmitter placement sequences). We calculate the optimal line barrier length for all different transmitter placement order, and find that the length of barriers is within the interval (1740,1741). 

In Figure 20, we experimentally obtain that the barrier length ranges from (1740,1741). The maximum and minimum values are due to the different placement order of transmitters, which involves the different placement order of receivers and the different placement intervals under different placement order of transmitters. We find that the differences of optimal coverage length between different transmitter deployment orders on the line barrier is less than 0.036%, so we deduce that when constructing a line barrier with heterogeneous BR sensors, the effect of the placement order of transmitters on the length of the longest line barrier can be neglected.

**Figure 21:**
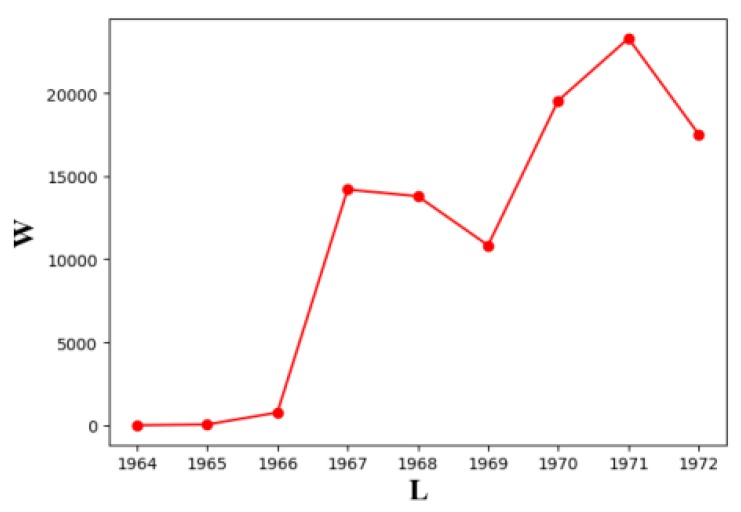
The influence of different placement sequences of heterogeneous transmitter on the length of heterogeneous line barrier (We use 6 types of transmitters, the number of 6 types of transmitters are {3,2,5,3,4,3} respectively, 20 transmitters in total, and test on 10,000 heterogeneous BR placement sequences).

We further perform the simulation experiments by changing the number of sensors. Similarly, we use six types of transmitters described above, but change the number of transmitters, the number of transmitters in each order is {3,2,5,3,4,3}, 20 transmitters in total, with 100 receivers. We conduct this experiment in the same way as above, but considering the whole sequence number of twenty transmitters is too large, we chose the typical ten sequences of transmitter as follows:

{*T_1_*, *T_1_*, *T_1_*, *T_2_*, *T_2_*, *T_3_*, *T_3_*, *T_3_*, *T_3_*, *T_3_*, *T_4_*, *T_4_*, *T_4_*, *T_5_*, *T_5_*, *T_5_*, *T_5_*, *T_6_*, *T_6_*, *T_6_*},

{*T_5_*, *T_6_*, *T_1_*, *T_3_*, *T_4_*, *T_5_*, *T_3_*, *T_6_*, *T_2_*, *T_3_*, *T_3_*, *T_1_*, *T_6_*, *T_2_*, *T_4_*, *T_5_*, *T_4_*, *T_3_*, *T_1_*, *T_5_*},

{*T_2_*, *T_5_*, *T_6_*, *T_1_*, *T_3_*, *T_2_*, *T_6_*, *T_5_*, *T_4_*, *T_4_*, *T_3_*, *T_1_*, *T_1_*, *T_3_*, *T_3_*, *T_3_*, *T_4_*, *T_5_*, *T_5_*, *T_6_*},

{*T_2_*, *T_4_*, *T_6_*, *T_3_*, *T_5_*, *T_1_*, *T_6_*, *T_5_*, *T_2_*, *T_3_*, *T_4_*, *T_1_*, *T_5_*, *T_3_*, *T_6_*, *T_5_*, *T_4_*, *T_3_*, *T_3_*, *T_1_*},

{*T_3_*, *T_5_*, *T_4_*, *T_2_*, *T_6_*, *T_1_*, *T_3_*, *T_5_*, *T_4_*, *T_2_*, *T_5_*, *T_6_*, *T_1_*, *T_1_*, *T_5_*, *T_4_*, *T_6_*, *T_3_*, *T_3_*, *T_3_*},

{*T_4_*, *T_1_*, *T_6_*, *T_2_*, *T_3_*, *T_5_*, *T_5_*, *T_1_*, *T_6_*, *T_2_*, *T_3_*, *T_3_*, *T_5_*, *T_1_*, *T_3_*, *T_3_*, *T_4_*, *T_5_*, *T_4_*, *T_6_*},

{*T_3_*, *T_4_*, *T_5_*, *T_1_*, *T_6_*, *T_2_*, *T_3_*, *T_6_*, *T_1_*, *T_4_*, *T_6_*, *T_5_*, *T_2_*, *T_1_*, *T_3_*, *T_4_*, *T_5_*, *T_3_*, *T_5_*, *T_3_*},

{*T_4_*, *T_5_*, *T_6_*, *T_1_*, *T_3_*, *T_4_*, *T_5_*, *T_2_*, *T_2_*, *T_1_*, *T_6_*, *T_6_*, *T_1_*, *T_3_*, *T_3_*, *T_3_*, *T_3_*, *T_4_*, *T_5_*, *T_5_*},

{*T_6_*, *T_6_*, *T_6_*, *T_5_*, *T_5_*, *T_5_*, *T_5_*, *T_4_*, *T_3_*, *T_4_*, *T_3_*, *T_4_*, *T_3_*, *T_3_*, *T_3_*, *T_2_*, *T_2_*, *T_1_*, *T_1_*, *T_1_*},

{*T_6_*, *T_5_*, *T_4_*, *T_3_*, *T_2_*, *T_1_*, *T_1_*, *T_2_*, *T_3_*, *T_4_*, *T_5_*, *T_6_*, *T_3_*, *T_4_*, *T_5_*, *T_6_*, *T_1_*, *T_3_*, *T_3_*, *T_5_*},

At the same time, the results of 10,000 sequences are calculated on the basis of each sequence, and 100,000 transmitter’s sequences in total, and then calculate the relation between *W* and the length of the longest barrier *L*, as shown in Figure 21. 

We find that the barrier length is stable in the interval (1964,1972), and the error of the optimal line barrier length is less than 0.5%. Different transmitter placement order has little effect on the optimal barrier length. In addition, we have carried out several similar simulations with different types of transmitters and different numbers of receivers, and we reach the same conclusion.

To sum up, to construct the longest heterogeneous line barrier under the condition of given SNR threshold and the set of heterogeneous BR transmitters and receivers, we propose an efficient approximate algorithm. First, for a given set of heterogeneous BR sensors, we randomly determine the location of transmitters, and then use Algorithm 2 in Section 4.2 to obtain the length of the longest line barrier and the optimal placement scheme. Up to this point we have solved Problem 2, and also Problem 1 through Algorithm 1.

## 5. Minimum Cost deployment of Heterogeneous Line Barrier

In this section, we apply the above optimal deployment method to solve the minimum cost placement problem of heterogeneous BR barrier in practical applications. The problem is presented as follow:

For *q* kinds of heterogeneous transmitters $T_{1}∼T_{q}$, the corresponding numbers of transmitters *M_1_* ~ *M_q_*, the numbers of receivers *N*, the unit price of *q* kinds of transmitter $C_{T_{1}}∼C_{T_{q}}$ and the unit price of the receiver $C_{R}$. How to construct the minimum cost deployment of heterogeneous BR sensor barrier with length *L* and satisfy with the predefined SNR threshold λ.

The minimum cost problem can be formulated as follows:

(7) $$\begin{array}{l} \mathrm{minimize}\;Cost\_T+Cost\_R\\ \mathrm{subject}\;\mathrm{to}\;\left\|P\right\|=L,V(P)\geq \lambda \end{array}$$

where *Cost_T* denotes the cost of transmitters and *Cost_R* denotes the cost of receivers. ‖P‖ denotes the length of the line barrier *P* and *V(P)* denotes the vulnerability of the barrier *P*. 

For the construction of minimum coverage cost heterogeneous sensor barrier with SNR threshold, we only need to consider the unit cost of transmitter and receiver based on Algorithm 2 that to solve approximate optimization detection performance of barrier. 

In Section 4.2, we have proposed the approximate optimal barrier placement algorithm. We apply these methods into the construction of minimum cost line barrier coverage with length *L*. Considering the coverage capability and unit price of different transmitters and receivers, a greedy algorithm for solving the minimum cost coverage problem of heterogeneous line barriers is proposed, as shown in Algorithm 3.

**Algorithm 3** Compute the minimum cost of line barrier33. 
$\mathrm{Input}:\;\bar{T}=\left\{\left\{T_{1}^{1},T_{1}^{2},...,T_{1}^{M_{1}}\right\},...,\left\{T_{q}^{1},T_{q}^{2},...,T_{q}^{M_{q}}\right\}\right\},\bar{R}=\left\{R_{1},R_{2},...,R_{N}\right\},L$
34. 
$\mathrm{Output}:\;\mathrm{the}\;\mathrm{minimum}\;\cos t\;C^{*},\;\mathrm{the}\;\mathrm{approximate}\;\mathrm{optimization}\;\mathrm{barrier}\;P^{*}$
35. 
for i←1:q 
36.  $\;\mathrm{For}\;j\leftarrow 1:C_{q}^{i}\;//C_{q}^{i}\;\mathrm{denotes}\;\mathrm{selecting}\;i\;\mathrm{transmitters}\;\mathrm{from}\;q\;\mathrm{transmitters}$37.   $\;\mathrm{Generate}\;a\;\mathrm{transmitter}\;\mathrm{set}\;S_{T}^{j}\;\mathrm{which}\;\mathrm{the}\;\mathrm{set}\;\mathrm{size}\;\mathrm{is}\;i\;\mathrm{and}\;\mathrm{one}\;\mathrm{transmitter}\;\mathrm{for}\;\mathrm{each}\;\mathrm{class}$38.   $P\leftarrow \mathrm{Algorithm}-2(\bar{T},\bar{R})$39.   C←Get_cost(P) //Compute the current minimum cost C40.   $\;\mathrm{If}\;C<C^{*}$41.    $C^{*}\leftarrow C;P^{*}\leftarrow P$42.    End If43.   End For44. 
End For
45. 
$\mathrm{Return}\;(C^{*},P^{*})$


First, we enumerate different numbers of transmitter combinations. According to the conclusions in Section 4.2.3, we do not need considering the transmitter placement order, which can greatly reduce the complexity of the algorithm. Then we consider the cost of the transmitter and receiver and use Algorithm 2 to calculate the current minimum coverage cost. Finally, the coverage cost of all transmitter combinations is compared, and the minimum coverage cost of the line barrier is obtained. 

## 6. Simulation Experiments

In this section, we use simulation experiments to verify the effectiveness of the proposed algorithms. We set up the parameters of heterogeneous BR sensor network, as show in Table 2. *L* denotes the length of the line barrier, and *K* denotes the physical coefficient of the transmitter. e denotes the absolute value of the difference of $S_{1}$, $S_{2}$, and $S_{3}$ barrier length. $C_{T}$, $C_{R}$ denote the unit price of transmitter and receiver, respectively. And $SNR^{*}$ denotes the optimal SNR for line barriers. The simulation experiments are performed on a 64-bit Windows 10 system. The algorithms are implemented in C++, and the visualization of placement results is implemented in Python. The software used is CodeBlocks and Pycharm.

In the simulation experiments, we set the length *L* of the line barrier from 100 to 200 with a step of 10, and the six types of transmitter is $T_{1}\sim T_{6}$, the value of *K* is {2,4,6,8,10,12}, respectively. The number of transmitters for each class is {3,2,5,3,4,3}, 20 in total, while the number of receivers is 100. 

**Figure 22:**
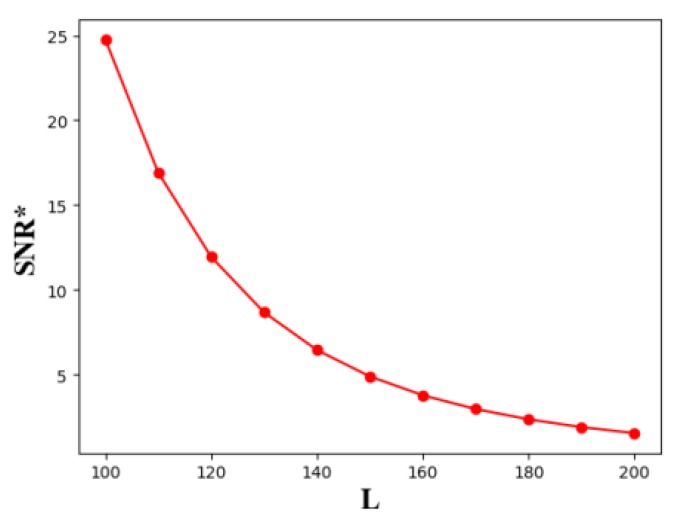
The relationship between line barrier length *L* and optimal $SNR^{*}$.

**Figure 23:**
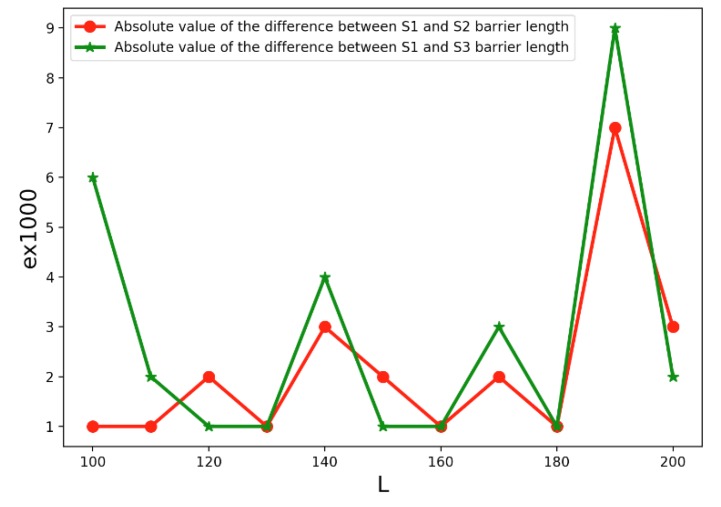
The Variation deviation of barrier length of difference placement orders: *S_1_*, *S_2_*, and *S_3._*

The following experimental results are calculated by using Algorithm 1 and Algorithm 2:

As shown in Figure 22, when the number of sensors is determined, with the decrease of the length of the line barrier, the optimal SNR threshold $SNR^{*}$ of the line barrier with the best monitoring performance is gradually increasing. At the same time, the smaller the barrier length, the faster the SNR increases. Then, we randomly obtain three placement sequences of transmitters:

*S_1_* = (*T_2_*, *T_5_*, *T_6_*, *T_1_*, *T_3_*, *T_2_*, *T_6_*, *T_5_*, *T_4_*, *T_4_*, *T_3_*, *T_1_*, *T_1_*, *T_3_*, *T_3_*, *T_3_*, *T_4_*, *T_5_*, *T_5_*, *T_6_*),

*S_2_* = (*T_4_*, *T_1_*, *T_1_*, *T_2_*, *T_2_*, *T_3_*, *T_3_*, *T_3_*, *T_3_*, *T_3_*, *T_4_*, *T_1_*, *T_4_*, *T_5_*, *T_5_*, *T_5_*, *T_5_*, *T_6_*, *T_6_*, *T_6_*),

*S_3_* = (*T_3_*, *T_1_*, *T_6_*, *T_2_*, *T_3_*, *T_5_*, *T_5_*, *T_1_*, *T_6_*, *T_2_*, *T_4_*, *T_3_*, *T_5_*, *T_1_*, *T_3_*, *T_3_*, *T_4_*, *T_5_*, *T_4_*, *T_6_*)

and conduct a comparison experiment, the results are shown in Figure 23.

We calculate the absolute value of variation deviation of *S_1_*, *S_2_*, and *S_3_* barrier length, separately. As shown in Figure 23, the absolute value of the difference is between 1000 and 10000.

At the same time, in most cases, the fluctuation of the difference is relatively stable. This feature further confirms our inference that the length of the longest line barrier has little relation with the heterogeneous transmitters’ placement order.

**Figure 24:**
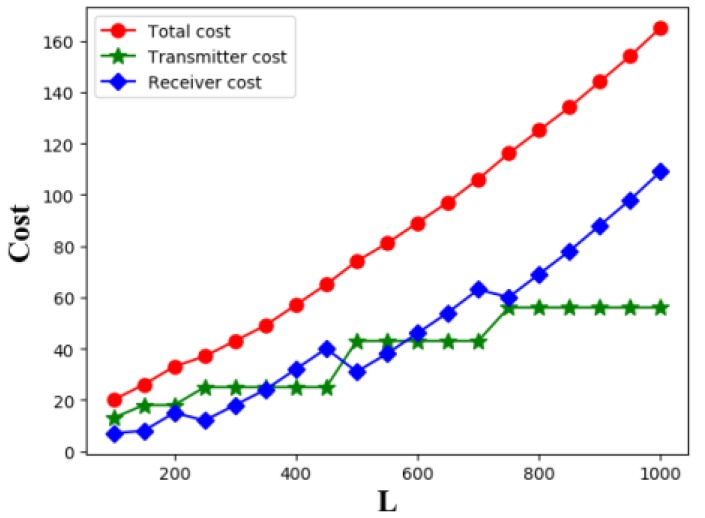
The relationship between line barrier length *L* and coverage cost.

Then, we consider the experiment of line barrier coverage with the minimum coverage cost. In this experiment, we set the length *L* of the line barrier from 100 to 1000 with a step of 50, and the six types of transmitter is $T_{1}\sim T_{6}$, the value of *K* is {2,6,9,13,18,25}, respectively. One for each kind of transmitters, while there is no limit to the number of receivers. The unit price of the transmitter $C_{T}$ is {2,6,9,13,18,25} and the unit price of receiver $C_{R}$ is 1. The following experimental results are calculated by using Algorithm 3, as shown in Figure 24.

**Figure 25:**
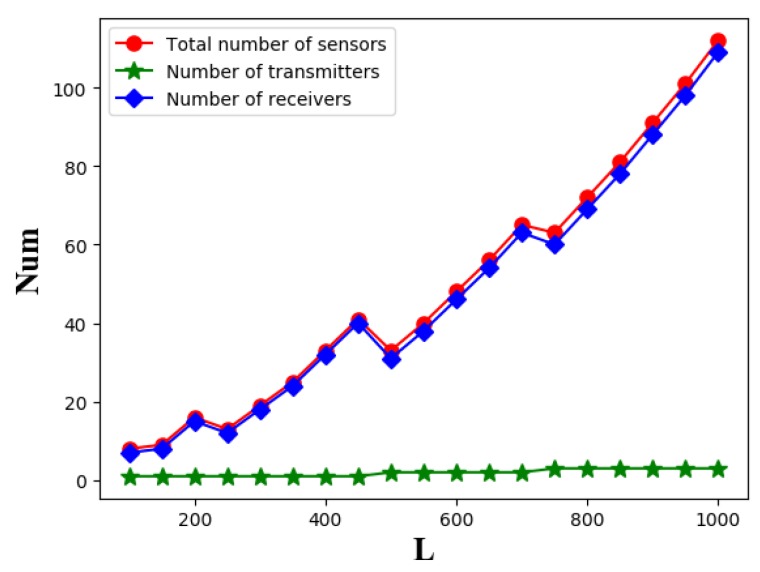
The relationship between the line barrier length *L* and the number of sensors.

Then, we consider the relationship between the number of sensors and the length *L* of line barrier, as shown in Figure 25. We find that as the length of line barrier increases, the total number of sensors appears to be wavy, but overall it is increasing. And the number of transmitters increased very slowly because the cost of transmitter was much more expensive than that of receiver. We find that the number of receivers also tends to be wavy, because as the length of line barrier increases, the optimal placement set of transmitter changes, causing the length of the coverage area of transmitter to change, making the required number of receivers changed. This conclusion reminds us that although receivers are cheaper, in some cases, choosing a slightly more expensive transmitter with strong coverage can reduce the receiver cost, thereby reducing the overall cost. For the line barriers of different lengths, we need to choose the right set of transmitters, which can reduce the cost of coverage.

## 7. Conclusions

In this paper, we study the optimal performance and minimum cost coverage of line barrier based on heterogeneous BR sensors. First, we consider the sensor optimal interval placement in homogeneous and heterogeneous sequences, respectively. Then, for the line barrier when the transmitter placement order is determined, we propose an optimization algorithm to construct the longest line barrier. Second, we consider the effect of different transmitter placement order on the length of line barrier, and through simulation experiments, we conclude that the transmitters’ sequence orders on the barrier have little influence on the variation of barrier’s length. Then, we propose an approximate optimal placement algorithm based on heterogeneous BR sensors to maximize the monitoring performance of line barrier. Finally, we consider the unit cost of transmitter and receiver, and propose a greedy algorithm for constructing line barrier coverage with minimum cost. For the future work, firstly, we will further study the factors affecting the placement of transmitter on the barrier. We will also consider the problem of belt barrier coverage based on heterogeneous BR sensors. Second, we will consider the BR sensors coverage problem on complex environment, such as underwater, underground and air. Third, we will consider coverage problem with different bistatic radar sensors, such as acoustic radar, electromagnetic radar and optical radar.

## Figures and Tables

**Table 1:** Parameter description.

Symbol	Quantity
$T_{i},\;R_{j},\;X$	BR transmitter *i*, BR receiver *j* and target
$\bar{T_{i}X}$	line segment between transmitter $T_{i}$ and target *X*
$\bar{R_{j}X}$	line segment between receiver $R_{j}$ and target *X*
$\left\|T_{i}X\right\|$	Distance between transmitter $T_{i}$ and target *X*
$\left\|R_{j}X\right\|$	Distance between receiver $R_{j}$ and target *X*
$T_{i}-R_{j}$	Pair of bistatic radar sensors
SNR(X)	Signal to noise ratio of target *X*
λ	SNR threshold of BR barrier
I(X)	Detectability of *X*
$I_{\max}$	Detectability threshold of BR barrier
$M_{1},\;...,\;M_{q}$	*q* kinds of BR transmitters and corresponding number of transmitters
$\bar{T}$	All transmitters
$\bar{R}$	All receivers
L	Line barrier length
V(P)	The vulnerability of barrier
$H_{l},\;H_{r}$	Left and right endpoint (node) of barrier
S	Placement sequence of BR sensors
$d_{i}$	placement spacing *i*
$d_{i}^{j}$	Placement spacing interval *i* of BR transmitter *j*
D	Maximum placement spacing sequence of internal coverage area
E	Maximum placement spacing sequence of external coverage area
$I_{\max}$	Dtectability threshold of BR barrier

**Table 2:** Parameter Setting.

Symbol	Quantity
*L*	(100,200),(100,1000)
*K*	{2,4,6,8,10,12}, {2,6,9,13,18,25}
*S_1_*, *S_2_*, *S_3_*	Transmitter placement order
*e*	The absolute value of the difference of *S_1_*, *S_2_*, and *S_3_* barrier length
*T_1_* ~ *T_6_*	Transmitter type
*C_T_*	{2,6,9,13,18,25}
*C_R_*	1
SNR^*^	The optimal SNR for line barriers

**Figure A1:**
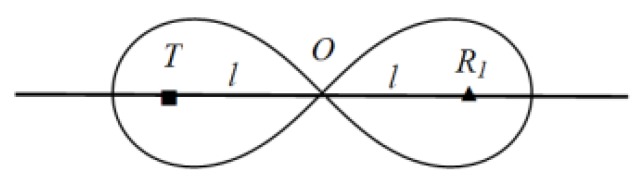
Maximum placement interval of $T-R^{n}$ when n=1. (Square denotes transmitter and triangle denotes receiver).

**Figure A2:**
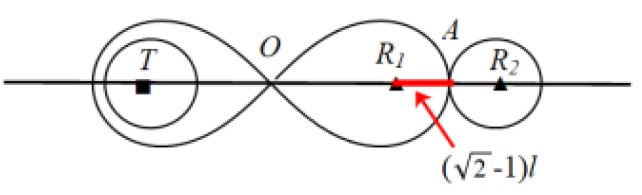
Maximum placement interval of $T-R^{n}$ when n=2 (The length of the red segment is $\frac{d_{2}}{2}=(\sqrt{2}-1)l$, where $d_{2}$ is the second maximum deployment interval).

**Figure A3:**
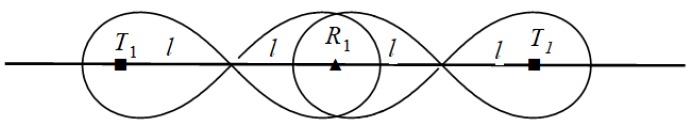
Maximum placement intervals of homogeneous BR sequence $T_{1}-R^{n}-T_{1}$ when n=1.

**Figure A4:**
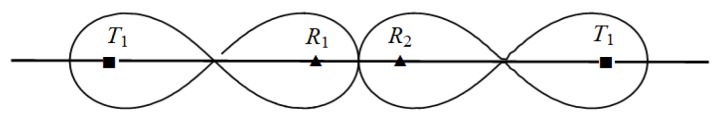
Maximum placement intervals of homogeneous BR sequence $T_{1}-R^{n}-T_{1}$ when n=2.

**Figure A5:**
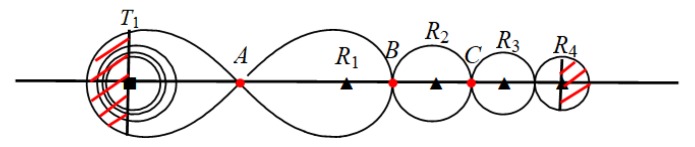
External coverage area of $T-R^{n}$.

**Figure A6:**
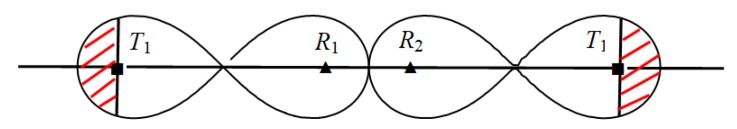
External coverage area of homogeneous BR sequence $T_{1}-R^{n}-T_{1}$.

## Appendix

**Proof of Theorem** **2.** According to Theorem 1, we find that the maximum detectability is at the midpoint of the segment which connects transmitter and receiver, and in order to meet the requirements of line barrier coverage, we only need to ensure that the detectability of each point on the barrier is not higher than $I_{\max}$. So according to Theorem 1, we have the following placement method: When n=1, as shown in Figure A1.

At this point, for the straight line segment between transmitter T and receiver $R_{1}$, the detectability of midpoint O is maximal, so in order to meet the coverage conditions, ensure the overall detectability of barrier is not higher than $I_{\max}$ and maximize the length of barrier, we only need to make $I(O)=I_{\max}$. So we have $\left\|TO\right\|\cdot \left\|R_{1}O\right\|=l^{2}$, that is $\left\|TO\right\|=\left\|R_{1}O\right\|=l$. Assume that the length of line segment $\bar{TR_{1}}$ is $d_{1}$, hawse have $d_{1}=2l$.

When n=2, as shown in Figure A2.

At this point, we will place receiver $R_{2}$ on the right side of receiver $R_{1}$. By the placement method of n=1, we can get the detectability of point A, that is $I_{\max}$. At the same time, according to the characteristics of the bistatic radar sensor model, when the distance between the transmitter and the receiver is greater than 2l, the coverage area is two spaces separated from each other which is similar to circle. Therefore, in order to get the maximum placement spacing of line barrier, we still ensure that the detectability of point A is $I_{\max}$. In order to ensure the continuity of barrier coverage, we use the placement method shown in Figure 6. Assume that the length of line segment $\bar{R_{1}R_{2}}$ is $d_{2}$, because of $\left\|TA\right\|\cdot \left\|R_{1}A\right\|=\left\|TA\right\|\cdot \left\|R_{2}A\right\|=I_{\max}$, then $\left\|R_{1}A\right\|=\left\|R_{2}A\right\|$. Assuming that $\left\|R_{1}A\right\|=x$, then $x\cdot (x+2l)=l^{2}$, solving the quadratic equation we get $x=(\sqrt{2}-1)l$, that is $\left\|R_{1}A\right\|=\left\|R_{2}A\right\|=(\sqrt{2}-1)l$, $d_{2}=2(\sqrt{2}-1)l$.

When n=k, we can get the equation $\frac{1}{2}d_{k}\cdot (\frac{1}{2}d_{k}+\sum_{i=1}^{k-1}d_{i})=l^{2}$, which calculates the length of line segment $\bar{R_{k-1}R_{k}}$. Combined with $d_{1},d_{2},...,d_{k-1}$, we can get $d_{k}=2(\sqrt{k}-\sqrt{k-1})l$. □

So, we get Theorem 2.

**Proof of Theorem** **3.** For placement sequence $T_{1}-R^{n}-T_{1}$, We also discuss the different cases in which the number of receivers ranges from 1 to *n*. When n=1, as shown in Figure A3.

Similar to Proof of Theorem 2, we found that when $\left\|T_{1}R_{1}\right\|=d_{1}=2l$, the line range of sensor coverage is largest.

When n=2, as shown in Figure A4.

We found that when $\left\|T_{1}R_{1}\right\|=d_{1}$ and $\left\|R_{1}R_{2}\right\|=d_{2}$, the sensor coverage on line barrier is longest.

We found that the covering length of the sequence $T_{1}-R^{n}-T_{1}$ is symmetrical, so we only need to discuss the parity of n.

When *n* is even, two transmitters divide the receiver set equally. Therefore, the maximum placement spacing sequence is $D=\left\{d_{1},d_{2},...,d_{(n+1)/2},d_{(n+1)/2},...,d_{2},d_{1}\right\}$.

When *n* is odd, the middle receiver will be shared by the two transmitters, so we can get the maximum placement spacing sequence is $D=\left\{d_{1},d_{2},...,d_{n/2+1},...,d_{2},d_{1}\right\}$. □

So we get Theorem 3.

**Proof of Theorem** **4.** The sequence $T-R^{n}$ represents placing n receivers on one side of transmitter T. As shown in Figure A5, the red shadows are the external coverage areas of sequence $T-R^{n}$.

According to the proof of the previous theorem, the coverage area of bistatic radar sensor is surrounded by Cassini’s oval line, so it is symmetrical and we can get $\left\|T_{1}A\right\|=\left\|R_{1}A\right\|$. Similarly, the length of the left shadow coverage region is equal to the length of line segment $\bar{R_{1}B}$, we set the length of the left external coverage area is $EL=\frac{d_{2}}{2}$. With respect to the length of the right shadow coverage region, we can obtain $\left\|R_{1}B\right\|=\left\|R_{2}B\right\|=\frac{d_{2}}{2}$, $\left\|R_{2}C\right\|=\left\|R_{3}C\right\|=\frac{d_{3}}{2}$ sequentially, according to the concept of detectability. According to the proof of Theorem 2, when the number of receivers is n, we can get the length of right shadow coverage region of sequence $T-R^{n}$, and we set the length of the right shadow external coverage area is $ER=\frac{d_{n+1}}{2}$. □

So, we get Theorem 4.

**Proof of Theorem** **5.** Similar to BR sequence $T-R^{n}$, the maximum placement spacing sequence in external coverage area of $T_{1}-R^{n}-T_{1}$ is calculated as shown in Figure A6.

In Figure A6, we deploy two receivers. We can find that continuously increasing the number of receivers between two transmitters which has the same properties will not affect the length of coverage area in shadow area, as shown in Figure 10. At the same time, we found that the coverage area of $T_{1}-R^{n}-T_{1}$ has axis symmetry, so the length of its left and right shadow external coverage areas is equal. We can get the length of the shadow external coverage area of placement sequence $T_{1}-R^{n}-T_{1}$ is $E=(EL,ER)=(\frac{d_{2}}{2},\frac{d_{2}}{2})$. □

So, we get Theorem 5.
